# Estimation of between-Cow Variability in Nutrient Digestion of Lactating Dairy Cows Fed Corn-Based Diets

**DOI:** 10.3390/ani10081363

**Published:** 2020-08-06

**Authors:** Himali Tharangani, Changwen Lu, Liansheng Zhao, Lu Ma, Xusheng Guo, William P. Weiss, Dengpan Bu

**Affiliations:** 1 State Key Laboratory of Animal Nutrition, Institute of Animal Science, Chinese Academy of Agricultural Sciences, Beijing 100193, China; htharangani@gmail.com (H.T.); luchangwen@sinofarm.com.cn (C.L.); aaronann@163.com (L.Z.); malu.nmg@163.com (L.M.); 2 State Key Laboratory of Grassland Agro-ecosystems, School of Life Sciences, Lanzhou University, Lanzhou 730000, China; guoxsh07@lzu.edu.cn; 3 Department of Animal Sciences, Ohio Agricultural Research and Development Center, Ohio State University, Wooster, OH 44691, USA; weiss.6@osu.edu; 4 Joint Laboratory on Integrated Crop-Tree-Livestock Systems of the Chinese Academy of Agricultural Sciences (CAAS), Ethiopian Institute of Agricultural Research (EIAR) and World Agroforestry Center (ICRAF), Beijing 100193, China

**Keywords:** sample size, variation, dairy cow, statistical power

## Abstract

**Simple Summary:**

Cow variability present in nutrient digestibility studies differs for different diets and nutrients. It is a major factor determining adequate sample size so that studies are not under-powered or over-powered. The objective of the current study was to develop cow variability estimates that can be used to determine the optimal sample size for digestibility trials having randomized block designs using mid-lactation dairy cows when fed corn-based diets having different neutral detergent fiber:starch ratio (0.7, 1.0, and 1.3). Cow variability is greater for digestibility of fiber and dry matter and less for starch. Estimated cow variability as standard deviations for digestibility of dry matter, neutral detergent fiber and starch were 3.8 g/kg, 5.1 g/kg and 3.3 g/kg, respectively. A major implication of this study is that cow variability is greatest for fiber digestibility and the use of a minimum of 12 cows per dietary treatment is adequate to reliably detect treatment effects on the digestibility of fiber, starch and dry matter using lactating dairy cows fed in groups with randomized block design under current experimental conditions.

**Abstract:**

The objective of this study was to estimate cow variability that can be used to determine the optimal sample size for digestibility trials using lactating dairy cows. Experimental design was randomized complete block design having three blocks and three dietary treatments. Three similarly managed nearby intensive farms were considered as blocks, and three diets were formulated to have 0.7, 1.0, and 1.3 neutral detergent fiber (NDF): starch ratio. In each farm, 18 cows were assigned for each dietary treatment and five sample sizes per each treatment group were simulated by simple random sampling of data from 18, 15, 12, 9 and 6 cows respectively. Intake was not affected by diet or sample size (*p* > 0.05). Estimated cow variability (as standard deviation) for digestibility of dry matter, NDF and starch were 3.8 g/kg, 5.1 g/kg and 3.3 g/kg, respectively. A major implication of this study is that cow variability is greatest for NDF digestibility and the use of a minimum of 12 cows per dietary treatment is adequate to reliably detect treatment effects on the digestibility of NDF, starch and dry matter using cows fed in groups with randomized block design under these experimental conditions.

## 1. Introduction

The gold standard for determining nutrient digestibility in dairy cows is direct measurement under production conditions and is not completely replaceable by metrics such as in vitro or in situ procedures [1]. However, the accuracy of in vivo values themselves depends on the experimental design, sample size, and experimental procedures [2,3]. Accurate detection of treatment differences in nutrient digestibility can be difficult because large between-animal variability requires large sample sizes [4]. Sample size requirements are affected by experimental design and generally, larger sample sizes are needed from randomized block designs than for Latin square experiments [5]. Using more animals than required statistically brings no added research value, but may lead to animal welfare concerns (excessive unnecessary use) and increase research costs without added value. Using an inadequate number of animals can reduce the accuracy of treatment means and prevent detecting treatment differences [6].

Power analysis is the most scientifically favored approach to determine the appropriate sample size [7]. The size of the treatment effect, the significance level, directionality of the test, and variability of experimental units as standard deviation (SD) are needed to conduct a power test to determine the required sample size. A major drawback of this method is that the sample size depends on the estimate of SD. This value is not available as the study has not yet been done; thus, it must be adapted from prior studies reported in the literature [8,9]. Unfortunately, those estimates can vary markedly between different studies due to the differences in factors such as DM (Dry Matter) intake, alteration of digestion and mean retention time in the rumen, changes in the rumen microbial population, anatomical differences and eating behavior.

For dairy cow nutrient digestibility experiments with completely randomized designs, few between-cow variability estimates for dry matter digestibility (DMD), NDF digestibility (NDFD), and starch digestibility (STRD) are reported in the literature. In one study, between-cow variation for DMD was higher (SD = 4.24 g/kg) than NDFD (SD = 2.82 g/kg) when cows fed diets with or without yeast (Saccharomyces cerevisiae) fermented-cassava chips when the experiment was designed in agreement with randomized complete block design (RCBD) [10]. In another study having RCBD, low between-cow variation for organic matter digestibility (SD = 2.25 g/kg) and high variation for NDFD (SD = 3.83 g/kg) were reported [2]. Further, lower between-cow variability for NDFD (SD = 1.61 g/kg) than DMD (SD = 2.05 g/kg) for a lactating dairy cow experiment having RCBD design also reported [11]. The contradictory nature of available literature data makes it difficult to get good estimates of variability to be used in statistical power analysis. The main objective of the current study was to develop between cow-variability estimates for the determination of optimal sample size for randomized block experiments so that differences in nutrient digestibility and lactation performance can be detected reliably. The hypothesis was that the sample size needed to evaluate treatment effects on nutrient digestibility of different diets in mid lactating dairy cows would be affected by diet composition.

## 2. Materials and Methods 

### 2.1. Experimental Design, Animals and Diets

All experimental procedures and animal use protocols were approved by the Animal Care and Use Committee of the Institute of Animal Science, Chinese Academy of Agricultural Sciences, Beijing, China (No. IAS20180115). The experimental design was a randomized complete block design. A digestibility trial was composed of three blocks and three dietary treatments. Three similarly managed intensive dairy farms (500 to 5000 cows) within close proximity of each other in northern China were used and considered as blocks [12]. Three experimental diets were formulated to result in three different NDF:starch ratios (0.7, 1.0, and 1.3) using corn silage as the only forage with different combinations of concentrates following NRC (2001) to meet the requirements of a 630–kg lactating dairy cow producing 42 kg of milk/d [13]. Ingredients and nutrient composition of dietary treatments are presented in Table 1. Corn silage produced at one farm was used in formulated diets for the whole experiment, and blocks (farms) executed the experimental protocol in parallel to maintain consistency of corn silage across farms. All the other ingredients were purchased from the same source and maintained in each farm separately. 

The sample size for the feed trial was projected at the planning stage using PROC MIXED procedure of SAS (SAS Institute Inc., Cary, NC, USA) using the PARMS statement. Anticipated mean NDF digestibility for three treatment groups (i.e., 45, 42 and 40), α = 0.05 and power = 0.80 were used to determine the appropriate sample size. Cow within a block, pen and treatment was considered as the experimental unit and referring to the prior studies, an SD of 3.1 was used as the between-cow variability. The projected sample size was nine cows per treatment. The resulted sample size was doubled and the experiment was conducted assigning 18 cows per treatment and it was assumed that the digestibility measured with 18 cows was adequate to detect treatment differences reliably. In each farm, 54 multiparous Holstein lactating dairy cows (parity, 3 ± 1; body weight, 605 ± 10 kg and days in milk, 150 ± 15) were randomly assigned to three pens in one enclosed barn having individual feeding compartments to have 18 cows in each pen. Three dietary treatments were assigned randomly across pens on each farm. The length of each feeding trial was 28 d and during the experiment, cows were fed twice per day at 0600 and 1600 h for 5% refusals. Milking was done twice daily at 0500 and 1400 h and individual daily milk yield (kg/d) of cows was recorded electronically. 

### 2.2. Measurements and Sample Collection

Daily individual DMI was determined by measuring TMR provided (DM basis) and subtracting the orts remaining (DM basis). Cow weights were recorded on d 1 and 28 in each trial, and initial body weight (BW) of cows was averaged 572 kg (±23 kg). During the feeding trial, TMR samples (500 g each) and orts (200 g) were collected daily (frozen at −20 °C) and composited weekly. Six fecal grab samples (~200 g of feces each) from each cow were collected twice daily to cover 0400, 0800, 1200, 1600, 2000, and 2400 h time points over final 3 days as previously described [14,15] and composited by wet weight for each cow over three days. 

### 2.3. Laboratory Analysis of TMR, Orts and Fecal Samples

Composited samples were dried at 60 °C for 48 h in a forced-air oven and were ground through a 1–mm screen (Wiley Mill, Arthur A. Thomas, Philadelphia, PA, USA). Ground samples were analyzed in duplicate for DM by oven-drying at 105 °C for three hours (method 930.15, AOAC, 2006), ash (method Ash 942.05; AOAC 2006), CP (method 990.03; AOAC, 2006), ether extract (method 2003.05; AOAC, 2006) [16] and NDF with an ANKOM 200 fiber analyzer (ANKOM Technology Corp., Fairport, NY, USA), with α-amylase and sodium sulfite (both from ANKOM Technology Corp., Fairport, NY, USA) [17]. Starch content of corn silage was determined enzymatically with a Megazyme Total Starch Assay Kit (Megazyme International Ireland Ltd., Bray, Ireland). 

Indigestible NDF (iNDF) of diets, feces and orts was obtained as previously described [18] by weighing samples (0.5 g) in to filter bags (25–µm pore size; Ankom Technology Corp.) and incubating in the rumen of two cannulated Holstein lactating dairy cows for 288 h. The cows were fed D2 (Table 1) twice daily at 0600 and 1600 h. At the end of incubation, bags were washed with abundant water until the water ran clear and then bags were analyzed for NDF (Ankom200 Fiber Analyzer; Ankom Technology Corp., Fairport, NY, USA) with heat-stable α-amylase and sodium sulfite. DMD, NDFD and STRD were calculated using iNDF and nutrient concentrations in the orts-adjusted diet and feces using the following equations:

DMD (% of DM intake) = 100 − {100 ∗ [iNDF] Diet/[iNDF] Feces)}(1)

NDFD (% of NDF intake) = 100 − 100 ∗ {[iNDF] Diet/[iNDF] Feces} ∗ {[NDF] Feces/[NDF] Diet}(2)

STRD (% of starch intake) was calculated the same way except [starch] replaced [NDF].(3)

### 2.4. Statistical Analysis

Initial assignment of 18 cows per treatment was used as the reference and based on the initial statistical power calculation, it was assumed as adequate to reliably detect treatment differences (>0.80 statistical power and *p* ≤ 0.05 significance level). Five sample sizes per each treatment group; N18 (data from all 18 cows), N15 (data from 15 cows), N12 (data from 12 cows) N9 (data from nine cows) and N6 (data from six cows) were simulated by simple random sampling of data from18 cows. Then, 1000 replicates for the five sample sizes were simulated by the bootstrapping method using PROC SURVEYSELECT procedure of SAS with strata specified as each treatment group [19]. Mean, median, variance and 95% confidence interval (CI) estimates were obtained for the original five sample sizes and bootstrap replicates using PROC UNIVARIATE statement of SAS and statistical power respective to each sample size also determined using PROC MIXED procedure of SAS using PARMS statement (α = 0.05, anticipated treatment means for D1, D2 and D3 were 45, 42 and 40, respectively).One-way ANOVA was conducted to compare bootstrap means respective to five sample sizes in each dietary treatment and mean differences were detected using the Tukey adjustment. The effect of diet on dry matter intake, nutrient digestibility and lactation performance of lactating dairy cows were tested using PROC MIXED procedure of SAS. The statistical model included the fixed effect of diet and random effect of the block and cow within the diet and farm. Pre-planned orthogonal contrasts were used to test for linear and quadratic effects of diet using the CONTRAST statement. This procedure was repeated five times to test the dietary treatment differences using five sample size groups. In addition, variance components associated with nutrient digestibility between-farms and within-farms were estimated using PROC MIXED procedure of SAS and the model included the random effects of farm, cow and the residual term. Statistical significance and trends were declared to be significant at *p* ≤ 0.05 and a trend when *p* > 0.05 to *p* ≤ 0.10, respectively. 

## 3. Results

### 3.1. Descriptive Statistics and Statistical Power Estimates of Original Five Sample Sizes and Bootstrap Replicates 

Descriptive statistics and statistical power estimates of the original five sample sizes and bootstrap replicates are reported in Table 2. Mean DMD, NDFD, STRD, variances respective to nutrient digestibility, and statistical power respective to different sample sizes of the bootstrap procedure were similar to the parameter estimates of five original sample sizes. Further, estimates of the original sample sizes fell within the 95% confidence interval obtained for the bootstrap replicates. 

### 3.2. Effect of Diet on Body Weight and DMI of Dairy Cows Fed Corn-Based Diets 

The effect of the experimental diet on body weight of cows and DMI are reported in Table 3. Body weight and dry matter intake of cows were not affected by dietary treatments and averaged 20.4 kg/d and 3.33 %BW (*p* > 0.05) when using data from all 18 cows. Mean body weight and DMI of three diets did not changed with reducing sample size from 18 to 6 per treatment (*p* > 0.05).

### 3.3. Effect of Diet on Nutrient Digestibility of Dairy Cows Fed Corn-Based Diets 

The effect of the experimental diet on digestibility of DM, NDF and starch in lactating dairy cows are reported in Table 4. DMD of diets decreased linearly with increasing NDF:starch ratio (*p* = 0.01) while STRD increased linearly (*p* = 0.04). The digestibility of NDF was not affected by diet (*p* = 0.07) when using data from all 18 cows. Mean DMD, NDFD, and STRD were not different when reducing sample size from 18 to 12. However, further reduction of sample size from 12 to 9 and 6 altered mean digestibility of three digestion variables (*p* ≤ 0.05).

### 3.4. Effect of Diet on Lactation Performance of Dairy Cows Fed Corn-Based Diets

The effect of the experimental diet on milk yield, contents and production of milk protein and fat in lactating dairy cows are reported in Table 5. When data from 18 cows are used, actual daily milk yield (kg/d) and 4% FCM yield (kg/d) were not affected by diet; however, milk fat % increased linearly with increasing NDF:starch ratio while decreasing milk protein content (*p* ≤ 0.05). Production of milk fat and milk protein were not different among dietary treatments (*p* > 0.05). The mean actual daily milk yield was different among five sample size groups (*p* ≤ 0.05) and according to the results, the mean actual milk yield in N18, N15 and N12 were not different in all three dietary treatment groups. However, reducing the sample size from 12 to 9 and further, milk yields changed. 4% FCM yield and the milk composition were not different among five sample size groups (*p* > 0.05). 

### 3.5. Effect of Cow Variability on Sample Size Determination and the Statistical Power Change with the Sample Size

**Figure 1:**
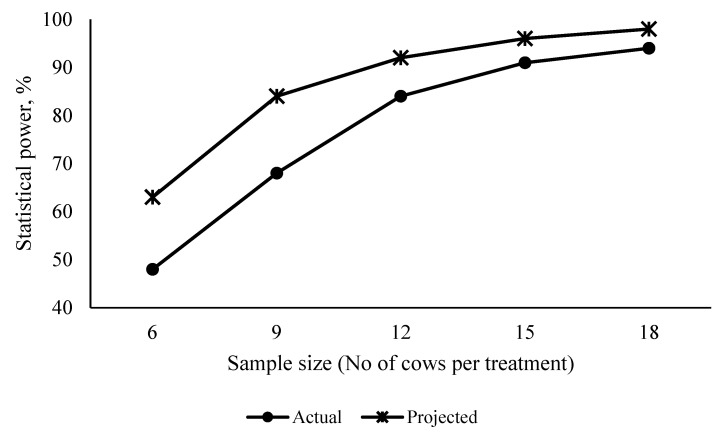
Plot of statistical power vs sample size. Projected and actual statistical power for different samples sizes that calculated at an alpha level 0.05. Anticipated treatment variability from the literature data were used in projecting statistical power at the research planning stage while actual treatment variability in nutrient digestion were used to determine actual statistical power at each sample size.

Projected and actual statistical power decreased when reducing sample size (*p* ≤ 0.05). Observed SD values for DMD, NDFD, and STRD ranged from 3.8 to 6.3, 5.1 to 9.3 and 3.3 to 5.6 respectively among different sample size groups (Figure 1) and those were higher than the SD value (3.1) used in statistical power analysis to determine the optimal sample size at the research planning stage. Consequently, statistical power projections at different sample sizes were overestimated by approximately 20–30 percent at each sample size (Figure 1) when all other factors are constant. Statistical power projection based on the literature data suggested that reducing sample size from 18 to 9 did not affect the statistical power of the study and assignment of minimum nine cows per treatment as adequate to reliably detect treatment differences (Statistical power = 0.84, α = 0.05). However, statistical power estimated using cow variability estimates of the current experiment showed that the assignment of 9 cows per treatment was not adequate to reliably detect treatment differences (Statistical power = 0.68%, α = 0.05) (Figure 1). 

### 3.6. Variance Components Associated with Nutrient Digestibility between and Within-Farm

**Figure 2:**
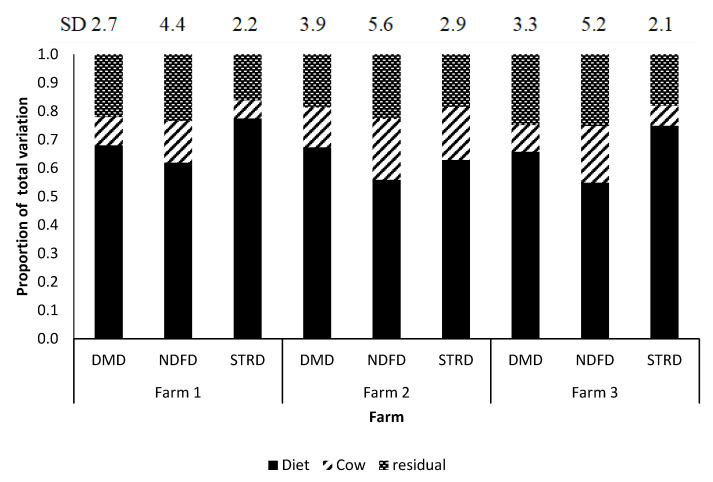
Variance components of nutrient digestibility estimates within farm. Variability are presented as proportion of total within farm variation. DMD = Mean and standard deviation (SD) of dry matter digestibility of cows; NDFD = Mean and standard deviation (SD) of NDF digestibility of cows estimated using iNDF as the internal marker; STRD = Mean and standard deviation (SD) of starch digestibility of cows estimated using iNDF as the internal marker.

Partitioning total variations (SD) in nutrient digestibility between and within-farms are presented in Table 6. The contribution of farm to the total variability in nutrient digestibility ranged 18–35%. As expected, the diet was the greatest contributor to the observed variability and accounted 40–62%. Between-cow variability usually comprised approximately 10% of the total variability in DMD, NDFD and STRD. Within-farm, the total variation in nutrient digestibility are presented as diet variance, between-cow variance, and residual components (Table 6) and Figure 2 shows the variance components as a proportion of the total within farm variation (n = 162). Diet was the primary source of variation for all digestibility variables within-farm and it was consistent among farms (approximately 50–70% of total variation). However, between-cow variation was different among farms and highest variability observed in farm 2 (13–18% of total variation) followed by farm 3 (7–15% of total variation) and farm 1 (5–11% of total variation), respectively. Within-farm, NDFD was the most variable digestibility parameter irrespective of the diet followed by DMD and STRD, respectively.

## 4. Discussion

The accuracy of five sample sizes was tested using 1000 bootstrap replicates, and according to our results, the amount of uncertainty associated with an estimate was less regardless of random sampling events as mean digestion variables of original five sample sizes fell within 95% confidence interval estimates of bootstrap replicates. Bootstrapping is a statistical procedure that re-samples a single original dataset to create many simulated samples [20,21]. When combined with descriptive statistics analysis and statistical power calculation, bootstrap sampling allows the generation of a series of estimates for means, medians, variances, CI and statistical power for simulated samples which could be used to test equality and accuracy of estimates. In that way, our results indicated that simulated five sample sizes were appropriate in determining the variability of nutrient digestion.

In lactating dairy cows, DMI is driven by milk production, yet physical fill of the reticulorumen and metabolic-feedback factors could limit the intake [22,23]. Dry matter intake of cows can increase when starchy concentrates replace forage [24,25]. Increased DMI increases the passage rate of digesta and causes a dip in digestibility of nutrients [26,27]. However, both DMI as kg/d and DMI as %BW were not affected either by NDF:starch ratio of the diet or sample size in our study. This lack of effect may be explained by the similar fill effect of the reticulorumen, from the NDF digestibility of corn silage and concentrates. Alternate reasons for similar DMI observed in the current experiment include compensatory intake proportionated with the bodyweight of cows. One study reported that DMI was not affected by NDF:starch ratio of diets within 0.74–1.27 range when diets were formulated to have equal in situ NDF digestibility [28]. However, DMI as a percentage BW decreased with increasing starch content of diets. In another study, similar DMI was reported within NDF:starch range of 0.86–1.18, and intake was reduced with the further increase of NDF:starch ratio up to 2.34 [29]. 

The decrease in DMD with increasing NDF:starch ratio observed in the current experiment was mostly caused by the replacement of highly digestible ground corn and cornmeal with low digestible soy hulls. However, NDFD was not different across experimental diets. This could be mostly caused by associative effects of NDF digestibility of corn silage and concentrates on three diets. Apart from that, the diet includes compensatory NDF digestion in the hindgut, and lack of differences among diets in carbohydrate fractions could be other contributing factors for the observed similar DMI. Comparable to our results, one study reported that decreasing NDF:starch ratios from 1.24 to 0.74 did not affect the NDF digestibility of corn silage-based diets when confounding factors are reduced [28]. In another study, DMD and NDFD decreased linearly when increasing NDF:starch ratios from 0.86 to 2.34 [29]. Starch digestibility increment with increasing NDF:starch was mostly caused by the change of starch source. In the diet with the highest NDF:starch ratio, corn silage provided 390 g/kg of DM dietary starch but in the diet with the lowest NDF:starch ratio, corn silage provided 290 g/kg of DM dietary starch. This was in agreement with Beckman and Weiss that the replacement of byproducts for corn grain increased the dietary proportion of the dietary starch contribution of corn silage led to increasing STRD with increasing NDF:starch [28]. In contrast, Firkins et al. [30] found a negative relationship between STRD and NDF:starch ratio though no effect was reported by Zhao et al. [29].

Although actual milk yield and 4% FCM yield were not affected by dietary treatments in the current experiment, we observed an increase in milk fat content from D1 to D3. Increments in NDF concentrations of diets tend to surge the proportion of acetate in the rumen which serves as the main precursor for milk fat synthesis [31,32]. Thus, the increase of milk fat content with increasing NDF:starch ratio could partially be described by enhanced NDF contents at the expense of starch. This is in agreement with Zhao et al. [29] that milk fat concentration was increased linearly when increasing NDF:starch ratio of diets from 0.86 to 2.34 due to the surge of the molar proportion of acetate in the rumen. In another study, Jenkins and McGuire [33] also reported that the acetate and butyrate which are contributing from NDF serve as the major precursors to support milk fat synthesis. 

Decrease of milk protein content with increasing NDF:starch ratio in the current experiment could be partially described within the experimental conditions by low dietary rumen fermentable carbohydrate content resulting from the replacement of highly digestible ground corn and cornmeal with low digestible soy hulls. This is well documented in many previous studies that milk protein content could increase as grain replaced forage or fibrous byproducts to result in low NDF:starch ratio [29,34,35]. In contrast, Beckman and Weiss [28] reported that the milk protein contents and yields were not affected by NDF:starch ratio as far as intake of digestible energy not affected by the treatment. However, the increment and reduction of milk fat and milk protein contents could not be fully explainable within the current experimental conditions as rumen fermentation parameters, concentrations of rumen microbial protein and energy contents were not determined.

Estimated statistical power at five sample sizes contradicted initial statistical power projections at the research planning stage and was mostly caused by observed high between-cow variability. The statistical power calculation is the most scientifically favored method used in animal studies in determining an appropriate sample size before the study, which ensures high reliability of the conclusions [36,37]. However, lack of good cow variability estimates for digestibility studies having randomized block designs in the literature leads to over-estimation of statistical power at different sample sizes in the current experiment. This result was in agreement with Biau et al. [38] that the underestimation of variability at the time of planning, the sample size computed would be too small, and the study would be underpowered to the one desired. Thus, initially projected sample size (i.e., nine cows per treatment) was doubled, and the experiment was conducted by assigning 18 cows/treatment to assure adequate statistical power to obtain cow variability estimates in nutrient digestion. Although reducing sample size from 18 to 12 did not affect mean nutrient digestibility and milk production estimates as statistical power was adequate to reliably detect treatment differences, the between-cow variability was higher than expected. Thus, the assignment of cows less than 12 per treatment showed as not adequate to reliably detect treatment differences.

Diet was the greatest contributor to the observed total variability farm-to-farm as imposed by treatments. As a general pattern, the effect of between-cow variability was less for highly digestible nutrients (i.e., starch) and more for lower digestible nutrients among farms (i.e., NDF). The farm was a substantial source of variation for the observed between-cow variability for all three nutrient digestion variables and this indicates that the effect of between-cow variation in nutrient digestibility of corn silage-based diets is farm specific. Differences among between-cow variability of nutrient digestion farm to farm could cause by genetic differences among cow groups, farm-specific housing and management factors related to cow nutrition such as accessibility to water and water quality, feeding management and likely several other unidentified factors [39,40]. Thus, variability estimates obtained from a group of cows in a single farm probably will not accurately represent cow variability in determining sample size on a specific farm for a digestibility trial.

Observed between-cow variability in nutrient digestibility within-farm could be due to differences in DM intake, alteration of digestion and mean retention time in the rumen, changes in the rumen microbial population, anatomical differences, sorting behavior during intake and likely several other unidentified factors [41,42]. Though DMI was not different across diets in the current experiment, the latter reasons could be responsible for the observed between-cow variation. Interestingly, the observed differences in the nutritional composition of orts in the current experiment could partially explain between-cow variability; however, the variability explained by sorting behavior could not be quantified. 

The highest between-cow variability observed for NDFD could be partially explained by diet characteristics and changes of passage rate of NDF from the rumen. A previous study reported a slight negative relationship between NDF intake and NDFD (R^2^ = 0.30), and according to the author, the passage rate of NDF from the rumen increased with increasing NDF intake due to the substantial variation in NDFD between cows, irrespective of intake [43]. The digestibility of starch had the lowest cow to cow variability. Within a diet, the between-cow variation could be explained by the enzyme activity of the rumen fluid and that residence time of starch in the rumen [44]. The activities of amylase and protease enzymes of rumen fluid are greatly variable between cows and increased with higher starch diets and lower NDF from forage [45] or beet pulp [46]. Further, differences of ruminal and intestinal enzymatic activities, how well cows chewed and broke up corn silage kernels and size of cecum could be other possible contributing factors for the observed between-cow variability in starch digestion [47,48]. 

Further, sampling and sample analysis could affect digestibility estimates, but variations associated with sampling and sample analysis could not be removed in the current experiment. Thus, between-cow variation estimated across diets and farms reflects the sampling and analytical variation in addition to true between-cow variation. In our study, total tract nutrient digestibility was measured using iNDF as the internal marker and rectal grab samples were used to determine nutrient and marker concentration in feces. However, the sampling method (grab samples vs. total collection), sampling frequency and variations in the iNDF method could contribute to the over-estimation of the between-cow variability. Although the total collection of feces is the most accurate procedure in quantifying nutrient digestibility [49], we adapted the spot sampling technique to overcome practical difficulties such as sampling from large no of cows, time, special facilities and the high cost associated with the total collection. As marker concentration of feces is highly variable throughout a day [15,50] a proper representation of diurnal variation in fecal marker concentration should be achieved by sampling at multiple time points. The use of different spot sampling frequencies in a 24-h cycle has been well documented in the literature in estimating total-tract digestibility [18,51,52]. However, findings of a recent study suggested that six sampling events (every 4 h in a 24 h cycle) as the minimum adequate fecal spot sampling frequency to use iNDF as an internal marker when estimating total tract nutrient digestibility [15].

## 5. Conclusions

Substantial between-cow variation in nutrient digestibility exists for diets with different NDF:starch ratio when fed to groups of cows in different farms. However, for a specific dietary treatment arrangement within-farm, between-cow variation in digestibility is not necessarily partitioned the same for different diets and dietary nutrients. Between-cow variation within-farm was highest for NDFD, followed by DMD and STRD, respectively. Thus, evaluating a given dietary treatment effect on NDFD is a primary objective, the sample size will need to be greater (i.e., a minimum of 12 cows per treatment) than if the digestibility of other components is the primary objective when fed lactating dairy cows in groups with randomized block designs under the conditions of this experiment.

## Figures and Tables

**Table 1:** Ingredient and chemical composition of three diets used in the current experiment.

Variable		Diets ^1^	
	D1	D2	D3
Ingredient Composition, (g/kg of Dry Matter)			
Corn silage	426	426	426
Dry ground corn	277	273	233
Soy Bean Meal, 48% CP	212	206	215
Soy hulls	41	46	63
Whole cottonseed	12	18	32
Animal fat	1.8	1.7	1.4
Vitamin and Mineral Mixture	30.2	29.3	29.6
Chemical composition (g/kg of Dry Matter) DM	564	579	583
NDF	239	273	302
CP	171	166	162
Ether extract	41	43	42
Ash	56	62	64
iNDF	84.2	89.5	92.4
Starch	343	267	232
NDF:starch ratio	0.7	1.0	1.3

^1^ D1, D2 and D3 were experimental diets having 0.7, 1.0 and 1.3 NDF: starch ratios, respectively.

**Table 2:** Descriptive statistics and statistical power estimates of original five sample sizes and bootstrap replicates.

Variables ^1^	Sample Size ^3^												
	N18	N15			N12			N9			N6		
	M18	M15	Bootstrap Rep ^2^		M12	Bootstrap Rep		M9	Bootstrap Rep		M6	Bootstrap Rep	
			BSM	CI		BSM	CI		BSM	CI		BSM	CI
DMD	0.678	0.682	0.683	0.66–0.70	0.666	0.670	0.64–0.67	0.718	0.713	0.69–0.73	0.741	0.743	0.70–0.75
NDFD	0.452	0.461	0.464	0.44–0.47	0.449	0.451	0.42–0.48	0.488	0.491	0.45–0.51	0.496	0.494	0.46–0.52
STRD	0.966	0.941	0.943	0.90–0.96	0.952	0.953	0.91–0.97	0.893	0.901	0.87–0.92	0.902	0.901	0.88–0.95
VDMD	3.8	3.9	3.8	3.6–4.1	4.2	4.6	3.8–4.8	5.1	5.3	4.7–5.6	6.3	6.8	6.2–6.9
VNDFD	5.1	5.4	5.9	5.1–6.2	6.3	6.6	6.0–6.8	7.4	7.9	7.1–8.2	9.3	9.8	8.9–10.4
VSTRD	3.3	3.4	3.7	3.0–4.2	3.8	3.9	3.5–4.3	4.1	4.5	3.9–4.8	5.6	5.8	5.1–6.7
Power	0.98	0.92	0.92	0.90–0.93	0.89	0.88	0.86–0.92	0.84	0.83	0.81–0.89	0.63	0.61	0.58–0.64

^1^ Variables: DMD = In vivo dry matter digestibility coefficient, NDFD = In vivo neutral detergent fiber digestibility coefficient measured using iNDF as the internal marker, STRD = In vivo total tract starch digestibility coefficient measured using iNDF as the internal marker, VDMD = Variance of dry matter digestibility that calculated as the square root of the variance-component estimate (g/kg), VNDFD = Variance of neutral detergent digestibility that calculated as the square root of the variance-component estimate (g/kg), VSTRD = Variance of starch digestibility that calculated as the square root of the variance-component estimate (g/kg), Power = Estimates of statistical power at alpha level 0.05 using actual treatment observed variability for different sample sizes using PROC MIXED procedure of SAS (SAS Institute Inc., Cary, NC) using PARMS statement. M18, M15, M12, M9 and M6 were means of nutrient digestibility, variances, and statistical power data obtained for the original five sample sizes. ^2^ Bootstrap rep = Bootstrap replicates median (BSM), and 95% confidence interval (CI) for bootstrap replicates obtained by 1000 random re-sampling events. ^3^ Sample size = Five sample sizes (N18, 18 cows per dietary treatment; N15, 15 cows per dietary treatment; N12, 12 cows per dietary treatment; N9, nine cows per dietary treatment; N6, six cows per dietary treatment).

**Table 3:** Effect of diet on body weight and dry matter intake of lactating dairy cows fed corn-based diets in different sample size groups (*n* = 162).

Variable ^1^	Sample Size Group ^2^	Diet ^3^			SEM ^4^	*p*-Value ^5^	
		D1	D2	D3		L	Q
DMI, kg/d	N18	20.9	20.6	19.7	0.09	NS	NS
	N15	20.6	20.2	20.2	0.11	NS	NS
	N12	20.7	20.4	20.4	0.11	NS	NS
	N9	21.0	20.3	20.8	0.19	NS	NS
	N6	21.0	20.8	20.5	0.24	NS	NS
	Mean ± SD	20.8 ± 0.18	20.4 ± 0.24	20.3 ± 0.4			
DMI, %BW	N18	3.43	3.37	3.21	0.14	NS	NS
	N15	3.42	3.33	3.30	0.09	NS	NS
	N12	3.41	3.35	3.31	0.13	NS	NS
	N9	3.44	3.33	3.44	0.12	NS	NS
	N6	3.48	3.41	3.38	0.14	NS	NS
	Mean ± SD	3.43 ± 0.02	3.36 ± 0.03	3.33 ± 0.09			
BW, kg	N18	609	611	615	0.42	NS	NS
	N15	602	607	612	0.35	NS	NS
	N12	606	608	616	0.31	NS	NS
	N9	610	610	614	0.27	NS	NS
	N6	604	606	615	0.38	NS	NS
	Mean ± SD	606 ± 3.34	608 ± 2.07	614 ± 1.52			

^1^ Variables: DMI = Dry matter intake per day in kilograms (kg/d) and intake as percentage body weight (%BW). ^2^ Sample size groups, N18 = 18 cows per dietary treatment; N15 = 15 cows per dietary treatment; N12 = 12 cows per dietary treatment; N9 = 9 cows per dietary treatment and N6 = 6 cows per dietary treatment, mean and standard deviations (SD). ^3^ Diet = D1, D2 and D3 were experimental diets having 0.7, 1.0 and 1.3 NDF:starch ratios respectively. ^4^ SEM = Standard error of the mean. ^5^ Probability of a linear (L) or quadratic (Q) effect of diet on body weight and dry matter intake of experimental cows (*n* = 162), NS = *p* > 0.1.

**Table 4:** Effect of diet on nutrient digestibility of dairy cows fed corn-based diets in different sample size groups (*n* = 162).

Variable ^1^	Sample Size Group ^2^	Diet ^3^			SEM ^4^	*p*-Value ^5^	
		D1	D2	D3		L	Q
DMD	N18	0.726 ^aA^	0.703 ^bB^	0.678 ^cB^	0.89	0.01	NS
	N15	0.718 ^aA^	0.707 ^bB^	0.681 ^cB^	0.92	0.02	NS
	N12	0.721 ^aA^	0.701 ^bB^	0.679 ^cB^	0.94	0.04	NS
	N9	0.684 ^bB^	0.689 ^aC^	0.669 ^cC^	1.13	0.03	NS
	N6	0.671 ^cC^	0.721 ^aA^	0.691 ^bA^	1.76	0.04	NS
	Mean ± SD	0.704 ± 0.02	0.704 ± 0.01	0.670 ± 0.01			
NDFD	N18	0.447 ^C^	0.452 ^C^	0.454 ^C^	1.20	0.07	NS
	N15	0.451 ^C^	0.458 ^C^	0.451 ^C^	1.43	0.08	NS
	N12	0.452 ^C^	0.454 ^C^	0.455 ^C^	1.45	0.09	NS
	N9	0.472 ^aB^	0.469 ^bB^	0.468 ^bB^	2.12	0.04	NS
	N6	0.481 ^aA^	0.474 ^cA^	0.478 ^bA^	2.45	0.03	NS
	Mean ± SD	0.460 ± 0.01	0.46 ± 0.01	0.46 ± 0.01			
STRD	N18	0.916 ^cC^	0.932 ^B^	0.961 ^aA^	0.77	0.04	NS
	N15	0.921 ^cC^	0.929 ^B^	0.968 ^aA^	0.81	0.03	NS
	N12	0.911 ^cC^	0.936 ^B^	0.964 ^aA^	0.84	0.05	NS
	N9	0.938 ^bB^	0.915 ^cC^	0.948 ^aC^	0.94	0.04	NS
	N6	0.952 ^A^	0.949 ^A^	0.952	0.96	NS	NS
	Mean ± SD	0.921 ± 0.02	0.930 ± 0.01	0.95 ± 0.01			

^a–c^ Least squares means in the same row with different superscripts differ (*p* ≤ 0.05). ^A–C^ Bootstrap means in the same column with different superscripts differ (*p* ≤ 0.05). ^1^ Variables: DMD = In vivo dry matter digestibility coefficient, NDFD = In vivo neutral detergent fiber digestibility coefficient measured using iNDF (g/kg DM) as the internal marker, STRD = In vivo total tract starch digestibility coefficient measured using iNDF (g/kg of DM) as the internal marker. ^2^ Sample size groups, N18 = 18 cows per dietary treatment; N15 = 15 cows per dietary treatment; N12 = 12 cows per dietary treatment; N9 = 9 cows per dietary treatment and N6 = 6 cows per dietary treatment, mean and standard deviations (SD). ^3^ Diet = D1, D2 and D3 were experimental diets having 0.7, 1.0 and 1.3 NDF:starch ratios respectively. ^4^ SEM = Standard error of the mean. ^5^ Probability of a linear (L) or quadratic (Q) effect of diet on nutrient digestibility of experimental cows fed corn-based diets (*n* = 162), NS = *p* > 0.1.

**Table 5:** Effect of diet on milk yield and composition of dairy cows fed corn-based diets in different sample size groups (*n* = 162).

Variable ^1^	Sample Size Group ^2^	Diet ^3^			SEM ^4^	*p*-Value ^5^	
		D1	D2	D3		L	Q
Milk yield, kg/d	N18	32.7 ^B^	32.2 ^B^	31.8 ^B^	1.64	NS	NS
	N15	32.2 ^B^	32.5 ^B^	32.0 ^B^	1.87	NS	NS
	N12	32.9 ^B^	32.1 ^B^	31.9 ^B^	1.95	NS	NS
	N9	33.8 ^aA^	33.2 ^aA^	32.4 ^bA^	3.21	0.07	NS
	N6	31.8 ^B^	31.5 ^C^	31.8 ^B^	3.67	NS	NS
	Mean ± SD	32.7 ± 0.76	32.3 ± 0.62	31.9 ± 0.25			
4% FCM, kg/d	N18	30.0	31.0	31.2	0.65	NS	NS
	N15	30.3	31.1	31.6	0.72	NS	NS
	N12	30.7	31.0	30.9	0.69	NS	NS
	N9	29.2	29.9	31.5	0.89	NS	NS
	N6	31.0	31.0	31.0	0.17	NS	NS
	Mean ± SD	30.2 ± 0.7	30.8 ± 0.5	31.2 ± 0.3			
Milk fat, %	N18	3.46 ^c^	3.78 ^b^	3.89 ^a^	0.13	0.04	NS
	N15	3.42 ^c^	3.72 ^b^	3.85 ^a^	0.11	0.03	NS
	N12	3.45 ^c^	3.71 ^b^	3.80 ^a^	0.19	0.05	NS
	N9	3.46 ^b^	3.76 ^a^	3.76 ^a^	0.23	0.05	NS
	N6	3.47 ^b^	3.77 ^a^	3.79 ^a^	0.22	0.04	NS
	Mean ± SD	3.45 ± 0.02	3.74 ± 0.03	3.81 ± 0.05			
Milk fat (g/kg)	N18	1.13	1.21	1.23	0.08	NS	NS
	N15	1.10	1.20	1.23	0.09	NS	NS
	N12	1.13	1.19	1.21	0.05	NS	NS
	N9	1.16	1.24	1.21	0.09	NS	NS
	N6	1.11	1.18	1.20	1.08	NS	NS
	Mean ± SD	1.12 ± 0.02	1.20 ± 0.02	1.21 ± 0.01			
Milk protein, %	N18	3.20 ^c^	3.16 ^b^	3.02 ^a^	0.21	0.05	NS
	N15	3.22 ^c^	3.20 ^b^	3.07 ^a^	0.32	0.04	NS
	N12	3.19 ^c^	3.16 ^b^	3.04 ^a^	0.29	0.05	NS
	N9	3.21 ^b^	3.18 ^b^	3.01 ^a^	0.54	0.08	NS
	N6	3.21 ^b^	3.19 ^b^	3.06 ^a^	0.65	0.07	NS
	Mean ± SD	3.20 ± 0.01	3.17 ± 0.01	3.04 ± 0.03			
Milk protein (g/kg)	N18	1.04	1.01	0.96	0.05	NS	NS
	N15	1.03	1.04	0.98	0.04	NS	NS
	N12	1.04	1.01	0.96	0.03	NS	NS
	N9	1.08	1.05	0.97	0.09	NS	NS
	N6	1.02	1.00	0.97	1.23	NS	NS
	Mean ± SD	1.04 ± 0.02	1.02 ± 0.02	0.96 ± 0.01			

^a–c^ Least squares means in the same row with different superscripts differ (*p* ≤ 0.05). ^A–C^ Bootstrap means in the same column with different superscripts differ (*p* ≤ 0.05). ^1^ Variables: Milk yield as kg per day, 4% FCM = 4% fat corrected milk yield (kg/d), contents (%) and production (g/kg) of milk fat and protein. ^2^ Sample size groups, N18=18 cows per dietary treatment; N15 = 15 cows per dietary treatment; N12 = 12 cows per dietary treatment; N9 = 9 cows per dietary treatment and N6 = 6 cows per dietary treatment, mean and standard deviations (SD). ^3^ Diet = D1, D2 and D3 were experimental diets having 0.7, 1.0 and 1.3 NDF:starch ratios respectively. ^4^ SEM = Standard error of the mean. ^5^ Probability of a linear (L) or quadratic (Q) effect of diet on lactation performance of experimental cows fed corn-based diets (*n* = 162), NS = *p* > 0.1.

**Table 6:** Variance component estimates (SD) for digestion variables of lactating dairy cows when fed experimental diets.

Item	Total Variability (SD, g/kg)				Within-Farm Variability (SD, g/kg)		
	Farm	Diet	Between-Cow	Residual ^1^	Diet	Between-Cow	Residual
DMD ^2^	2.31	3.15	0.48	0.73	4.76	1.85	2.72
NDFD ^3^	3.46	4.25	1.12	1.76	6.37	3.62	4.07
STRD ^4^	1.32	4.72	0.62	0.94	6.65	2.63	3.3

^1^ Residual SD includes within-cow variability for digestion variables. ^2^ DMD = Mean and standard deviation (SD) of dry matter digestibility of cows. ^3^ NDFD = Mean and standard deviation (SD) of NDF digestibility of cows estimated using iNDF as the internal marker. ^4^ STRD = Mean and standard deviation (SD) of starch digestibility of cows estimated using iNDF as the internal marker.
